# Cardiac Amyloidosis Mimicking Heart Failure With Preserved Ejection Fraction: Evaluating Self-Reported Red Flags in Routine Clinical Practice

**DOI:** 10.7759/cureus.90659

**Published:** 2025-08-21

**Authors:** Adil Mushtaq, Anum Faiz, Nayyar Iqbal Tiwana, Pankit Wadhwa, Sara Zubair Ahmed, Azmir Ali Khan, Roukaya Lamine Hamadi, Maryam Sabah, Hafsa Aslam, Ahmad Maher Husni Abdelkhalik, Saifullah Syed, Zainab Salahuddin

**Affiliations:** 1 Internal Medicine, Akhtar Saeed Medical and Dental College, Lahore, PAK; 2 Cardiology Medicine, Jinnah Hospital Lahore, Lahore, PAK; 3 Internal Medicine, Muzaffarnagar Medical College, Meerut, IND; 4 Internal Medicine, Baqai Medical and Dental University, Karachi, PAK; 5 Medicine, King Edward Medical University, Lahore, PAK; 6 Internal Medicine, Dubai Medical College, Dubai, ARE; 7 Internal Medicine/Cardiology, Dow University of Health Sciences, Karachi, PAK; 8 Internal Medicine, Fujairah Hospital, Abu Dhabi, ARE; 9 Internal Medicine, Federal Medical College, Islamabad, PAK; 10 Internal Medicine, Tbilisi State Medical University, Tbilisi, GEO; 11 Medicine, Royal College of Surgeons in Ireland, Dublin, IRL; 12 College of Medicine, Shaukat Khanum Memorial Cancer Hospital and Research Centre, Lahore, PAK

**Keywords:** cardiac amyloidosis, hfpef, nyha class, quality of life, red flag symptoms, screening

## Abstract

Background

Cardiac amyloidosis is often underdiagnosed and can mimic heart failure with preserved ejection fraction (HFpEF), particularly in older adults. Most cases of cardiac amyloidosis are discovered in the late stages as a result of overlapping clinical symptoms. This study addresses whether patients with symptomatic heart failure and associated red flag features of cardiac amyloidosis have a worse functional status and quality of life than patients with classical HFpEF.

Methodology

This cross-sectional study was conducted between July 2024 and May 2025, in Lahore, Pakistan, in the cardiology departments of hospitals and clinics. Participants were recruited using a convenience method, in which 389 adults experiencing heart failure symptoms were interviewed. Data were collected using a structured, self-reported questionnaire that included demographic items, a seven-item checklist of red flag symptoms (e.g., carpal tunnel syndrome, unintentional weight loss, dizziness, and unexplained shortness of breath), and validated tools of physical limitations and quality of life. To assess the association between red flag symptoms and clinical outcomes, non-parametric statistical tests, correlation tests, and ordinal logistic regression were employed using SPSS Version 26 (IBM Corp., Armonk, NY, USA).

Results

Among 389 participants (mean age 51.4 ± 8.8 years), those with two or more red flags (suspected cardiac amyloidosis group) had significantly worse NYHA functional class scores (U = 954.500, p < 0.001) and lower quality of life scores (U = 580.000, p < 0.001) compared to typical HFpEF patients. The suspected cardiac amyloidosis group had a median New York Heart Association (NYHA) score of 3.00 (interquartile range (IQR) = 3.00-4.00) and a median quality of life score of 42.00 (IQR = 32.00-54.00). The red flag burden was positively correlated with worse functional status (r = 0.26, p < 0.01) and negatively correlated with quality of life (r = -0.32, p < 0.01). Logistic regression showed that poorer functional status and quality of life were significant predictors of suspected cardiac amyloidosis classification.

Conclusions

The results showed that an increase in the burden of red flag symptoms is linked to greater physical limitation and poorer quality of life among heart failure patients. Although no diagnostic imaging was conducted in this study, the symptom-based screening tool can complement echocardiographic vigilance in clinical practice, helping identify individuals at risk for cardiac amyloidosis who may benefit from further investigations. Further studies are necessary to validate these tools and analyze the ways of implementing them within the early referral process.

## Introduction

Heart failure with preserved ejection fraction (HFpEF) has emerged as a significant clinical concern, with symptoms often overlapping with those of cardiac amyloidosis. Key red flags, such as carpal tunnel syndrome, unexplained weight loss, and cardiac hypertrophy, are commonly seen in conditions such as transthyretin familial amyloid polyneuropathy, a form of cardiac amyloidosis [1,2]. HFpEF is a heterogeneous syndrome characterized by diastolic dysfunction, poor systolic reserve, and systemic and peripheral disturbances, despite a normal ejection fraction [1]. HFpEF occurs in about half of heart failure patients and is linked with significant morbidity and mortality. The rising awareness has led to the stimulation of new research on the complex pathophysiology and therapeutic issues in HFpEF [3].

HFpEF has a substantial burden worldwide that involves more than 30 million individuals with annual mortality rates of between 10% and 30%. Sudden cardiac death and heart failure are typical causes of death, and diagnosis and treatment involve such instruments as the H2FPEF score, sodium-glucose co-transporter 2 inhibitors, diuretics, and lifestyle interventions [4,5]. The prevalence of preserved ejection fraction (HFpEF) was approximately one-third of individuals diagnosed with heart failure, and clinical outcomes, such as one-year mortality and readmission rates, were comparable between HFpEF and reduced ejection fraction patients. Patients with HFpEF were older, more often female, and had hypertension or atrial fibrillation more frequently. Despite the increasing recognition of HFpEF, its diagnosis remains challenging due to the nonspecific symptoms and overlap with other cardiovascular diseases, including heart failure with reduced ejection fraction. This often leads to delayed diagnosis and management, contributing to uncertain outcomes for patients [6].

Cardiac amyloidosis, an infiltrative cardiomyopathy caused by the accumulation of extracellular proteins, is a crucial prognostic feature in systemic amyloidosis [7]. The prevalence of cardiac amyloidosis as a cause of HFpEF is also being increasingly recognized, frequently associated with high medication intolerance and poor prognosis. The absence of screening of patients chosen to participate in the trial on amyloidosis in HFpEF may be the reason behind the inefficiency of therapy, and justifies putting in place regular methods of screening [8].

HFpEF is a growing challenge to older people, which is characterized by high readmission and mortality rates. A significant cause of HFpEF, although underdiagnosed, is transthyretin amyloidosis, especially senile forms, which should be considered in patients with infiltrative patterns on heart imaging and early screening, followed by genetic analysis and amyloid type should be performed to guide successful early therapy [9,10]. Myocardial fibrosis, hypertrophy, and inflammation were prevalent in a prospective HFpEF cohort with cardiac amyloidosis in 14% of patients, almost half of whom were previously unrecognized. These data indicate the importance of tissue analysis in enhancing diagnostic accuracy and directing therapy in HFpEF [11]. Cardiac imaging plays a crucial role in the assessment of heart failure and, more specifically, HFpEF, to detect possible underlying pathologies and inform adequate interventions. Echocardiography and cardiac MRI provide mutually complementary advantages in determining the structure, functions, and properties of myocardial tissue [12].

Rationale

Cardiac amyloidosis is a relatively neglected disease that may closely resemble HFpEF due to its clinical presentation. Patients can have typical HFpEF symptoms, namely, fatigue, shortness of breath, and exercise intolerance, whereas the actual cause can be missed, such as amyloid deposition in the cardiac muscle. Due to the similarity of the two conditions in early clinical presentation, most instances of cardiac amyloidosis remain misdiagnosed or are diagnosed at late stages, thus restricting the ability to intervene early and affecting outcomes.

A key limitation in the routine evaluation of HFpEF is the lack of screening for cardiac amyloidosis. Amyloidosis is not included in most HFpEF diagnostic protocols, leading to the condition being missed or delayed. This lack of standard screening measures is one of the reasons why the diagnosis and management of cardiac amyloidosis are delayed.

This study aims to raise awareness of symptom patterns that may be associated with suspected cardiac amyloidosis in patients with HFpEF-like symptoms, using a simple, non-invasive symptom-based tool to assist in early clinical suspicion, albeit not with diagnostic precision. The study employs structured observational scales without special tests to evaluate routine findings that are already known in everyday practice. This aims to boost awareness and recognition of patterns that might otherwise be missed, resulting in increased suspicion and earlier referral for confirmatory assessment in case of need.

Objectives

Primary Objective

This study aimed to assess the difference in physical limitations and quality of life between heart failure patients with a higher number of red flag signs of cardiac amyloidosis and those with typical HFpEF, as measured by a symptom-based, self-reported checklist.

Secondary Objectives

The secondary objectives were to categorize heart failure patients based on the number of red flag symptoms reported and assess their functional status and quality of life, using the symptom-based self-reported checklist. In addition, we aimed to compare the physical limitations and symptom burden between patients with few red flag symptoms versus those with multiple red flag symptoms. Finally, we aimed to evaluate the feasibility and effectiveness of a symptom-based screening tool in identifying patients at an increased risk of poor health outcomes.

## Materials and methods

This cross-sectional study was conducted from July 2024 to May 2025 to determine whether patients with heart failure and more red flag symptoms indicative of cardiac amyloidosis had worse physical health limitations and quality of life. Participants were adults with the symptoms of heart failure who were identified in the cardiology departments of hospitals and clinics in Lahore, Pakistan. Patients had no prior diagnosis of a particular type of heart failure; their grouping was based on a structured red flag symptom checklist administered during the research.

For data collection, structured questionnaires were used to measure demographic characteristics, symptom burden, physical limitations, and quality of life. The participants answered these questionnaires themselves, although they were helped where necessary. No diagnostic or laboratory tests were performed in the study; instead, the nature of the work purely depended on self-reported information and structured symptom checklists. Participation was voluntary, and only those who provided informed consent were included. This observational method was chosen because it is non-invasive and emphasizes the potential role of symptom-based red flags in identifying high-risk individuals during ordinary clinical practice without resorting to sophisticated diagnostic means. A convenience sampling method was employed due to resource constraints and feasibility within the context of healthcare settings in Lahore, which allowed us to reach a broad pool of participants quickly.

Sample size and technique

The number of heart failure patients suspected of having cardiac amyloidosis in the general population cannot be estimated precisely. As such, the population was deemed to be infinite in line with this study. Sample size calculation was done based on the following formula: \[n = \frac{Z^2 \cdot p (1 - p)}{d^2}\]

In this equation, Z is the standard score required to achieve the desired level of confidence, P is the estimated proportion, based on the available literature, and d is the allowable margin of error. The confidence level was set at 95% with a Z of 1.96, obtained using a t-scoring table, and 0.05 was used as d. The value of p was estimated at 0.50 to guarantee the largest required sample size. Based on this approach, a sample size of 389 was determined to be minimal. Other participants were contacted to cover possible non-responses or half-filled questionnaires [13].

A convenience sampling method was employed in selecting respondents through the cardiology departments of hospitals and clinics in Lahore, Pakistan. This non-probability technique enabled the researchers to recruit individuals satisfying the inclusion criteria who were available and willing to do so at the time of data collection. While convenience sampling allowed for efficient participant recruitment, it introduces potential selection bias and limits the generalizability of the findings to the broader population. However, efforts were made to recruit participants from multiple healthcare sites to ensure a diverse sample within the local context. Table 1 summarizes the inclusion and exclusion criteria.

**Table 1:** Inclusion and exclusion criteria of the study participants. HFpEF: heart failure with preserved ejection fraction

Inclusion criteria	Exclusion criteria
Individuals aged 18 years and above	Patients with confirmed cardiac amyloidosis.
Appearing with heart failure symptoms (e.g., dyspnea, fatigue, etc.).	Patients with a reduced ejection fraction (i.e., not an HFpEF profile). A reduced ejection fraction was identified according to the recent echocardiogram reports included in the patients’ medical records, with the related ejection fraction being less than 40%. The echocardiogram data were required to be from within the last six months before participation in the study
Able and willing to sign informed consent	Individuals with pre-diagnosed structural heart diseases, independent of HFpEF
Cooperative and available to fill out the questionnaires	Unfinished questionnaire or withdrawal of permission

Data collection tools

A structured questionnaire was employed in this study, which comprised four major groups, i.e., demographic data (basic), red flag symptom screening, functional status, and quality of life evaluation. In addition to a self-derived screening checklist, standardized tools were also used to cover a large proportion of the variables related to heart failure and suspected cardiac amyloidosis adequately. Each of the instruments was administered in a self-report format, and an assistant was available to assist if necessary.

Demographic Information

The first section of the questionnaire collected demographic and clinical background to investigate the extent to which individual variables can affect functionality status and outcomes in terms of quality of life. The data gathered included information on factors such as age, gender, and the duration of symptoms related to the heart problem, as well as whether individuals had comorbid conditions and whether they used the prescribed medication for their heart problems. It also asked participants and their family members whether any of them had ever been diagnosed with HFpEF, and whether any of them had experienced heart disease, heart failure, or any sudden cardiac death before the age of 60 years. This section provided a detailed description of the study sample, offering the opportunity to make comparisons between subgroups regarding clinical and demographic characteristics relevant to cardiac health (Table 2).

**Table 2:** Demographic information questionnaire. HFpEF: heart failure with preserved ejection fraction

Items	Responses
Age	-
Gender	-
Duration of heart-related symptoms	-
Do you currently have any of the following conditions?	-
Are you currently taking any of the following medications for heart-related conditions?	-
Family history of heart disease or sudden death (before the age of 60 years)	-
Have you ever been told you have HFpEF	-
Have you undergone an echocardiogram (heart ultrasound) in the last 12 months?	-

Red Flag Symptom Checklist

The self-created red flag questionnaire was utilized to identify the possible predictors of cardiac amyloidosis in individuals with symptoms of heart failure. The instrument was composed of seven questions about the symptoms or clinical indicators that have been most frequently reported, e.g., “Have you experienced unintentional weight loss in the past 6 months?” and “Do you feel numbness, tingling, or burning sensations in your hands or feet?” All questions were answered with a simple “Yes” or “No,” and the sum of points allowed for dividing participants into the following two categories: 0-1 red flags (typical HFpEF) and two or more red flags (suspected cardiac amyloidosis). It should be noted that the tool was developed solely for screening and exploratory comparison of the scale. Therefore, it cannot be considered a diagnostic tool for cardiac amyloidosis (Table 3). Additionally, the checklist was developed in English, and no formal translation or cultural adaptation was conducted for the local population. Future studies may consider translating and validating this checklist in the local language for broader applicability.

**Table 3:** Screening questions: symptom red flags for cardiac amyloidosis.

Items	Responses
Have you ever been diagnosed with carpal tunnel syndrome (pain or numbness in hands/wrists)?	-
Have you experienced unintentional weight loss in the past six months (not due to diet or exercise)?	-
Do you often feel dizzy or faint when standing up from a sitting or lying position?	-
Do you feel numbness, tingling, or a burning sensation in your feet or hands (especially at rest or at night)?	-
Do you have frequent episodes of shortness of breath or palpitations (fast heartbeat) without a known cause?	-
Is there any family history of cardiac amyloidosis or unexplained heart failure or sudden death?	-
Has your doctor ever advised you to avoid blood pressure medications due to low blood pressure or fainting?	-

New York Heart Association Functional Classification

In the current study, the New York Heart Association (NYHA) Functional Classification was employed as a measure of the extent of physical disability among participants who exhibited symptoms of heart failure. It divides people into four functional classes (Class I to IV) depending on the extent to which their symptoms (e.g., breathlessness or fatigue) affect their ability to perform physical activity. Class I indicates no restriction in everyday life, whereas Class IV signifies severe symptoms even at rest. This tool was first developed in 1928 and remains one of the most widely accepted and straightforward methods for assessing functional capacity in patients with heart failure to this day. There is no numerical scalability; however, it can be clinician-rated or self-reported, and it utilizes standardized definitions. Although inherently subjective, the NYHA classification has demonstrated reasonably strong reliability for use in studies [14].

Kansas City Cardiomyopathy Questionnaire

The Kansas City Cardiomyopathy Questionnaire (KCCQ), the 12-item form, was employed to evaluate the perceived quality of life based on the presence of heart failure symptoms. The KCCQ was initially produced in 2000 by Dr. John A. Spertus and others at the Mid-America Heart Institute. The KCCQ-12 measures four areas, including physical limitation, frequency of symptoms, quality of life, and social limitation. The results of each item are rated on a scale of 0-100, and responses on a Likert scale are converted into a score, where the higher the score, the higher the health status. The scores calculated for the domain are then averaged to yield a summary score. The KCCQ-12 has demonstrated excellent internal consistency, with a reported Cronbach’s alpha of 0.89, indicating very high reliability. KCCQ-12 is self-reported and short. It was adopted in this study to measure the consequences of heart failure symptoms on the daily functioning and well-being of participants [15].

Procedure

The participants were recruited through the cardiology departments located in multiple hospitals and clinics across Lahore, Pakistan. Initially, approximately 420 participants were contacted to participate, with a final sample size of 389 participants included in the study. This sample size was determined after accounting for possible non-responses and incomplete questionnaires. We reached out to additional participants to cover potential non-responses, ensuring the final sample met the necessary statistical requirements. No pilot testing was conducted, but the questionnaires were thoroughly examined in terms of clarity and relevance under consultation with local healthcare practitioners to ensure they were clear and culturally suitable for the participants. The questionnaire was either self-administered or administered by trained data collectors as deemed necessary by the participants. The research questionnaire was in its original English-language format, and a local language version of the questionnaire was not adopted, but in case a respondent failed to understand or read the questions, they were offered assistance. To ensure accurate data collection, data collectors received thorough training on study procedures, questionnaire administration, and ethical guidelines. This training emphasized minimizing biases and ensuring that respondents’ answers were not influenced. Supervisory checks were implemented to ensure adherence to the data collection protocols. Confidentiality was maintained by stripping all identifying details before data entry, in consideration of ethical concerns. The procedure was conducted in a respectful manner, which permitted the involvement of people with diverse cultural, educational, and medical backgrounds, which contributed to inclusiveness and accuracy of data during the study.

Statistical analysis

Data analysis was conducted using SPSS Statistics version 26 (IBM Corp., Armonk, NY, USA). Demographic information about the participants, such as age, gender, and use of medications, among others, was described in terms of frequencies and percentages. The Red Flag Questionnaire, NYHA, and KCCQ were assessed for normality using the Kolmogorov-Smirnov and Shapiro-Wilk statistics. The results showed that the data were not normally distributed, with varying p-values on the normality tests. The relationships between the variables were then investigated using Spearman rank-order correlation analysis, where it was revealed that the correlation between Red Flag Questionnaire (diagnostic group) and NYHA (functional class) scores was significant, as well as the correlation between Red Flag Questionnaire (diagnostic group) and KCCQ (quality of life) scores. Additionally, the Mann-Whitney U test was used to compare the scores between the different diagnostic groups (typical HFpEF vs. suspected cardiac amyloidosis) and gender groups, with the results showing significant differences. The Kruskal-Wallis H test was applied to determine the difference in scores in response to the questionnaires, based on age group and recognition of the presence of HFpEF diagnosis, which was found to be significant in both cases. Ordinal logistic regression analysis was used to predict membership of the diagnostic groups using NYHA scores and KCCQ, where both variables were significant predictors. Data analysis was conducted as a probability test with p-values <0.05, and any missing data were treated with listwise deletion by counting only complete data in the analyses. Although we used listwise deletion to address the missing data, which can decrease the sample size, as well as lead to bias in the case that the missing data are not missing completely at random. Imputation techniques might be used in future research designs as the missing data may be handled better and the items within the sample size retained, subsequently, minimizing the chances of bias.

Ethical considerations

The study was conducted by established protocols for research involving human subjects. Before starting the study, the institutional review board (IRB-24-04) at the Shaukat Khanum Memorial Cancer Hospital and Research Centre in Lahore reviewed and approved the research protocol. This approval stipulated that the ethical principles of respect for persons, beneficence, and confidentiality were adhered to during the research process. The study was conducted in accordance with the Declaration of Helsinki, which outlines ethical principles for conducting research involving human participants, ensuring their safety, privacy, and informed consent. The individuals were all fully aware of the study’s aim, procedures, risks, and benefits involved. Each participant signed informed consent before data collection. The participation was voluntary, and participants had the right to withdraw from the study at any time without penalty. All the personally identifying information was then eliminated or anonymized to ensure anonymity. The data were stored on password-protected computers and in locked storage (where possible) and were accessible only to authorized staff of the research team. These safeguards ensured no violation of the privacy and dignity of the participants involved in the study.

## Results

Table 4 reveals gender differences in the diagnostic group, NYHA functional class, and KCCQ-12 quality of life scores. In the Red Flag Questionnaire (diagnostic group), females (mean rank = 226.84) had a significantly higher mean rank than their male counterparts (mean rank = 180.52), with a Mann-Whitney U value of 15,543.00 (p = 0.006) and a small effect size (r = 0.14). Females (mean rank = 229.26) were ranked higher than males (mean rank = 177.60) (U = 15,035.00, p = 0.001, r = 0.16) in the NYHA functional class. Similarly, the mean rank of males (211.47) was significantly higher than that of females (168.37) on the KCCQ-12 quality of life, with a U value of 14,862.00 (p < 0.001) and a moderate effect size (r = 0.17). These results suggest that gender differences influence both functional status and quality of life, and, in general, females exhibit a lower functional status and quality of life than males.

**Table 4:** Mann-Whitney U test comparing participants of both genders on diagnostic group, NYHA, and KCCQ-12 scores. N = number of participants; U = Mann-Whitney U statistic; Z = standardized test statistics; r = effect size (rank-biserial correlation); ** = p < 0.01 considered significant. NYHA = New York Heart Association; KCCQ-12 = Kansas City Cardiomyopathy Questionnaire-12

Variable	Gender	N	Mean rank	Sum of ranks	U	Z	P-value	r (effect size)
Red Flag Questionnaire (diagnostic group)	Male	234	180.52	42,781.68	15,543.00	–2.75	0.006^**^	0.14
	Female	155	226.84	35,423.32				
NYHA functional class	Male	234	177.60	41,958.40	15,035.00	–3.21	0.001^**^	0.16
	Female	155	229.26	35,746.60				
KCCQ-12 quality of life	Male	234	211.47	49,482.00	14,862.00	–3.38	<0.001^**^	0.17
	Female	155	168.37	26,223.00				

Table 5 shows the Kruskal-Wallis H test comparisons Red Flag Questionnaire (diagnostic group), NYHA functional class, and KCCQ-12 quality of life scores by age group. There were significant variations between the three variables, with the difference expressed as a significant age difference. In the Red Flag Questionnaire, the age group of 60+ years was found to be significantly lower in the scores (mean rank = 183.48) than the younger age groups (under 40, 40-49, and 50-59) with a Kruskal-Wallis H value of 13.305 (p = 0.004). In terms of NYHA functional class, Patients in the 60+-year age group were significantly worse in the functional state (mean rank = 260.49) than all other categories, with an H value of 41.260 (p < 0.001). In quality of life KCCQ-12, the age group of 60+ years showed significantly lower scores (mean rank = 156.47) than the 40-49-year and 50-59-year age groups, with an H value of 11.812 (p = 0.008). These results point to the worse functional status and quality of life of older participants (60+ years) in comparison to younger ones, illustrating the role of age in health outcomes associated with heart failure.

**Table 5:** Kruskal-Wallis H test comparing Red Flag, NYHA, and KCCQ-12 scores by age. N = number of participants; H(χ²) = Kruskal-Wallis test statistic; df = degree of freedom; IQR = interquartile range; † = pairwise differences determined by Dunn-Bonferroni post hoc tests; ** = p < 0.01 considered significant. NYHA = New York Heart Association; KCCQ-12 = Kansas City Cardiomyopathy Questionnaire-12

Variable	Age group	N	Mean ± SD	Median (IQR)	Mean rank	H(χ²)	df	P-value	Significant pairwise differences †
Red Flag Questionnaire (diagnostic group)	Under 40 years	13	15.20 ± 3.50	15 (13–18)	199.00	13.305	3	0.004**	60+ < Under 40, 60+ < 40–49, 60+ < 50–59
	40–49 years	173	14.95 ± 3.40	15 (13–17)	196.76				
	50–59 years	127	15.05 ± 3.45	15 (13–17)	197.47				
	60+ years	75	14.30 ± 3.20	14 (12–15)	183.48				
NYHA functional class	Under 40 years	13	2.20 ± 0.77	2 (2–3)	203.08	41.260	3	<0.001**	60+ > all other groups
	40–49 years	173	2.05 ± 0.71	2 (2–2)	168.43				
	50–59 years	128	2.30 ± 0.74	2 (2–3)	191.72				
	60+ years	75	2.85 ± 0.68	3 (3–3)	260.49				
KCCQ-12 quality of life	Under 40 years	13	64.10 ± 15.30	66 (54–77)	198.58	11.812	3	0.008**	60+ < 40–49, 60+ < 50–59
	40–49 years	173	66.80 ± 14.90	69 (57–78)	209.20				
	50–59 years	128	64.50 ± 15.10	65 (54–76)	198.02				
	60+ years	75	58.20 ± 14.40	57 (48–69)	156.47				

Table 6 shows the Kruskal-Wallis H test comparing Red Flag scores, NYHA functional class, and KCCQ scores in the three groups, according to the answers to the question whether HFpEF was diagnosed or not: Yes (N = 121-122), No (N = 136), and Not sure (N = 131). In the Red Flag Questionnaire, the difference between the groups was significant (H(2) = 8.712, p = 0.013). The mean ranks in the Yes group (175.40) were lower, and in the No (207.60) and Not sure (210.80) groups were higher, which means that people who did not know or were confused about any diagnosis had more red flag qualities that are indicators of the presence of cardiac amyloidosis. There was also a notable variation in NYHA functional class (H (2) = 10.264, p = 0.006), with the yes population once again recording a mean rank of lower than average (176.55) compared to the unaware and unsure populations. Likewise, the results were significant between groups regarding the KCCQ scores (H(2) = 9.598, p = 0.008), with respondents who were aware of their HFpEF diagnosis reporting the lowest quality of life (mean rank = 168.75) compared to those who answered No (214.30) and Not sure (196.40) with a significantly higher quality of life. These results indicate that a higher symptom load and lower functioning could accompany the realization or doubt about HFpEF diagnosis. Diagnosed patients reported a low overall quality of life.

**Table 6:** Kruskal-Wallis H test comparing Red Flag, NYHA, and KCCQ-12 scores by HFpEF diagnosis awareness. N = number of participants; H(χ²) = Kruskal-Wallis test statistic; df = degree of freedom; * = p < 0.05; ** = p < 0.01 considered significant. NYHA = New York Heart Association; KCCQ-12 = Kansas City Cardiomyopathy Questionnaire-12; HFpEF = heart failure with preserved ejection fraction

Variable	HFpEF diagnosis awareness	N	Mean rank	H(χ²)	df	P-value
Red Flag Questionnaire (diagnostic group)	Yes	121	175.40	8.712	2	0.013^*^
	No	136	207.60			
	Not sure	131	210.80			
NYHA (functional class)	Yes	122	176.55	10.264	2	0.006^**^
	No	136	213.20			
	Not sure	131	202.00			
KCCQ-12 (quality of life)	Yes	122	168.75	9.598	2	0.008^**^
	No	136	214.30			
	Not sure	131	196.40			

Table 7 shows that the predictors of diagnostic group membership between typical HFpEF and suspected cardiac amyloidosis were strong. The risk of suspected cardiac amyloidosis participants was also significant (B = 1.950, p < 0.001). The NYHA functional class also had significant predictive power with a positive coefficient (B = 0.580, p < 0.001) such that, as the NYHA functional score increased, there was a higher potential of falling among the suspected cardiac amyloidosis category. Conversely, the quality of life score provided by the KCCQ-12 had a negative coefficient (B = -0.065, p = 0.002), indicating that being identified with suspected cardiac amyloidosis is associated with worse quality of life scores. The model fit statistics showed that the model explained 26.8% of the variance (pseudo-R² = 0.268), with the likelihood ratio χ² statistic confirming overall model significance (χ² = 46.32, p < 0.001). The goodness-of-fit statistic and the test of parallel lines both indicated that the model fit the data adequately (p = 0.412 and p = 0.146, respectively).

**Table 7:** Ordinal logistic regression predicting diagnostic group membership (typical HFpEF vs. suspected cardiac amyloidosis) based on NYHA (functional class) and KCCQ-12 (quality of life) scores. DV = diagnostic group (1 = typical HFpEF; 2 = suspected cardiac amyloidosis); B = coefficient; SE = standard error; β = standardized coefficient; LL = lower limit; UL = upper limit; Cl = confidence interval; ** = p < 0.01 considered significant. NYHA = New York Heart Association; KCCQ-12 = Kansas City Cardiomyopathy Questionnaire-12; HFpEF = heart failure with preserved ejection fraction

Variable	B	95% Cl		S.E	β	P-value
		LL	UL			
Threshold (Group 2)	1.950	1.400	2.500	0.280	-	<0.001^**^
NYHA (functional class)	0.580	0.280	0.880	0.150	0.250	<0.001^**^
KCCQ-12 (quality of life)	-0.065	-0.105	-0.025	0.020	-0.31	0.002
Model fit statistics	Pseudo-R² (Nagelkerke)	Likelihood ratio χ² (df = 2)	Goodness-of-fit (Pearson χ²)	Test of parallel lines χ²(2)	-	-
	0.268	46.32, p < 0.001^**^	521.84, p = 0.412	3.85, p = 0.146	-	-

The diagnostic group membership (typical HFpEF vs. suspected cardiac amyloidosis) in this study was based on the number of red flag symptoms rather than a confirmed diagnosis of cardiac amyloidosis. The red flag symptom checklist served as an exploratory screening tool, and the findings reflect suspected cases of cardiac amyloidosis, not those confirmed through diagnostic tests such as cardiac MRI or biopsy.

**Figure 1:**
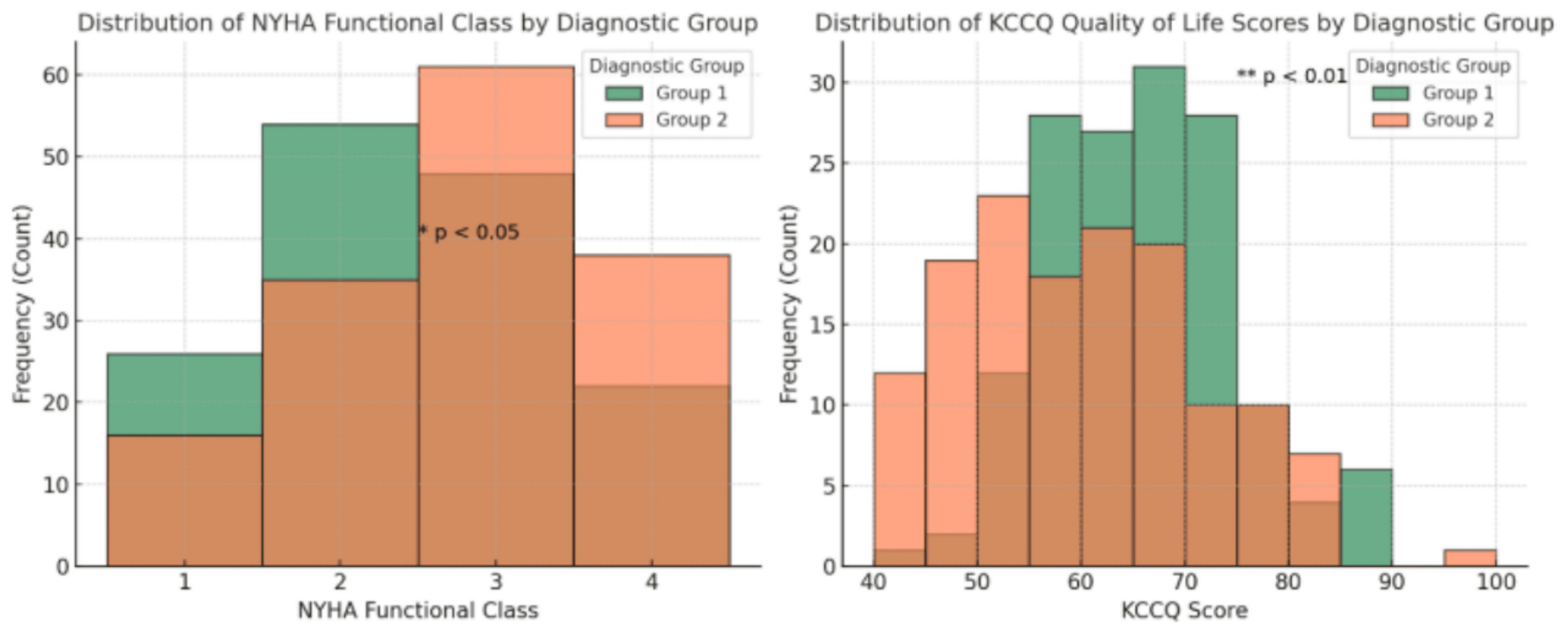
Histograms of NYHA (functional class) and KCCQ-12 (quality of life) scores by diagnostic group. NYHA = New York Heart Association; KCCQ-12 = Kansas City Cardiomyopathy Questionnaire-12

Figure 1 compares the distribution of New York Heart Association (NYHA) functional class and KCCQ quality of life scores between the two diagnostic groups. In the NYHA histogram (left), Group 1 (green) tends to cluster more in lower functional classes (1-2), indicating better cardiac function, while Group 2 (orange) shows a higher proportion in class 3, suggesting worse functional status; the difference is statistically significant (p < 0.05). In the KCCQ histogram (right), Group 1 generally has higher quality of life scores, with many patients scoring 65-80, whereas Group 2’s scores are shifted toward lower ranges (50-65); this difference is highly significant (p < 0.01), highlighting poorer perceived health status in Group 2.

Table 8 presents the proportion of heart-related medication usage by age group. Considerable variations were found between the age groups in the use of medications (χ² = 34.7, p < 0.001). The most frequently used drug was a beta-blocker, with the most significant prevalence in the 40-49-year group and the lowest in the under-40-year group. The use of diuretics was very similar, as 39 people were in the 40-49-year bracket and 33 in the 60+-year bracket. The highest frequency of angiotensin-converting enzyme inhibitors/angiotensin II receptor blockers and calcium channel blockers was observed in the group aged 40-49 years. The percentage of individuals not currently taking any medication was the highest in the 60+-year group and the lowest in the 40-49-year group. Such findings indicate different patterns of medication usage and age groups, with older age groups demonstrating increased medication use, especially when dealing with conditions such as high blood pressure and heart failure.

**Table 8:** Heart-related medication usage by age (N = 389). P-values calculated using the chi-square test; ** = p < 0.01 considered significant. ACE = angiotensin-converting enzyme; ARBs = angiotensin II receptor blockers

Age group	N	Beta-blockers	Diuretics	ACE inhibitors/ARBs	Calcium channel blockers	No current medication	df	P-value	χ²
Under 40 years	26	13	6	1	0	1	12	<0.001^**^	34.7
40–49 years	346	173	39	44	27	32			
50–59 years	256	128	34	37	18	12			
60 years and above	150	75	33	21	2	3			

Table 9 presents the family history of heart disease by age group. The chi-square test results (χ² = 13.4, p = 0.038) indicated a significant difference in the family history of heart disease across different age groups. Among the under-40-year-old group, two participants reported a family history of heart disease, while five reported no history, and six were unsure. The highest proportion of participants unsure about their family history was in the 60+-year group. In contrast, the 40-49-year group had the highest number of participants who confirmed a family history, while the 50-59-year group showed the highest proportion of participants with no family history. These findings suggest that awareness of family history of heart disease varies across age groups, with older individuals more likely to be uncertain about their family history, possibly due to a lack of knowledge or reporting.

**Table 9:** Family history of heart disease by age (N = 389). P-values calculated using the chi-square test; ** = p < 0.01 considered significant.

Age group	N	Yes	No	Not sure	df	P-value	χ²
Under 40 years	13	2	5	6	6	0.038^*^	13.4
40–49 years	173	37	55	81			
50–59 years	128	22	41	65			
60 years and above	75	9	13	53			

## Discussion

The current study aimed to investigate the clinical significance of red flags, such as carpal tunnel syndrome, unexplained weight loss, and cardiac hypertrophy, that may indicate cardiac amyloidosis in patients with heart failure. The study also aimed to analyze whether these red flags are linked to poor functional status and reduced quality of life. Our results suggest that higher red flag burden correlates with lower functional status and quality of life. This confirms past literature emphasizing that cardiac involvement in systemic amyloidosis forecasts adverse outcomes and that early recognition is critical to intervention [16]. Additionally, our study showed that higher functional limitation was significantly related to poor quality of life. This is consistent with prior studies that have documented significant changes in the quality of life of patients, most notably older adults, as physical functioning deteriorates over time [17].

Our study found that patients with suspected cardiac amyloidosis exhibited significantly worse functional class compared to those with typical HFpEF, reflecting greater functional impairment due to heart failure symptoms. This finding is consistent with previous research, which also highlighted the severe functional impairment associated with cardiac amyloidosis, particularly in terms of heart failure progression and its impact on overall functional status [18,19]. A recent study corroborates these findings, noting that transthyretin amyloid cardiomyopathy (ATTR-CM), a form of cardiac amyloidosis, is a progressive and life-threatening condition, leading to restrictive cardiomyopathy and heart failure as a result of the deposition of misfolded transthyretin protein in the myocardium [20]. Our study found that patients with suspected cardiac amyloidosis reported significantly worse quality of life compared to those with typical HFpEF. This aligns with previous studies, which also highlighted a substantial decline in quality of life in patients with cardiac amyloidosis, with patients experiencing notable impairments in general health and well-being [21,22]. Furthermore, diagnostic delays, common in ATTR-CM, exacerbate functional impairment and the decline in quality of life, underscoring the importance of early detection and timely intervention to improve outcomes and access to new disease-modifying therapies [23].

Although females in our study experienced a greater incidence of red flag symptomatology, the literature currently suggests a higher diagnostic rate of cardiac amyloidosis in men; this can be attributed to recent meta-analyses, such as the AS-CA meta-analysis [24]. This difference was found to be statistically significant in our sample, with females demonstrating higher red flag scores relative to males, due to a greater number of symptoms that may be predictive of cardiac amyloidosis. This disparity can be an indication of a diagnostic gap, whereby women presenting with early symptoms do not receive a vigorous checkup to determine the presence of cardiac amyloidosis. These findings underline the necessity of symptom-based screening tools to facilitate earlier detection, especially in women who are likely to be underdiagnosed. Women in our study had more physical limitations relative to men. This is in line with earlier reports regarding the functional status disparity between the genders, as women have a greater prevalence of physical impairment compared to men [25]. A review on gender differences in cardiovascular diseases further supported the concept of diagnostic gaps, noting that women may experience delays in diagnosis due to differences in clinical presentation and risk factors, highlighting the importance of considering gender-specific factors in clinical evaluations [26]. This difference in functional status was statistically significant according to our data, with women exhibiting a poorer functional status, as indicated by the ranks of the NYHA functional classes. Furthermore, our findings suggest that women with poor quality of life are supported by other studies, which affirm that women have a lower quality of life when physical and psychosocial factors are affected. Their health is negatively compromised [27]. In line with this, hormonal changes in postmenopausal women, such as variations in luteinizing hormone, sex hormone-binding globulin, and dehydroepiandrosterone, have been shown to significantly impact sexual function and various aspects of quality of life, including vitality and pain [28]. The disparity in quality of life between men and women in our sample was significant, supporting the notion of a gender-based difference in quality of life. Several potential confounding factors could influence these gender differences in symptom burden and functional status. Comorbidities such as hypertension, diabetes, or obesity may be more prevalent in one gender, affecting both the NYHA and KCCQ-12 scores. Moreover, treatment disparities, such as differences in medication adherence, access to care, or physician biases in diagnosis and treatment, could also contribute to these observed differences. These factors should be explored in future studies to better understand the underlying causes of the gender differences and inform more targeted diagnostic and treatment strategies.

Although the clinical status was more compromised, older adults in our investigation had slightly fewer red flags. This observation is reinforced by the results of previous studies that suggested that red flag symptoms can manifest years before diagnosis but have been neglected in elderly patients, delaying the diagnosis of cardiac amyloidosis [29]. Functional status decreased with increasing age, with the highest level of limitations reported by the older (60+ years) participants in our study. These results align with population-based research demonstrating an age-related decline in physical performance, accompanied by increased age-related functional limitations in older adults, particularly in women [30]. Hormonal changes, including a decrease in sex hormones such as estrogen and testosterone, contribute to this decline in physical performance and functionality, with studies showing that these hormonal shifts can exacerbate age-related limitations, especially in postmenopausal women [31]. We found that participants aged 60 years and above had significantly poorer scores on the quality of life measure compared to younger participants. However, there is some evidence in the literature that age in itself does not impact quality of life adversely when health and psychosocial aspects are well addressed, referring to the role of resilience and adaptation in old age [32].

In our study, the burden of symptoms was more significant, and functional scores were lower in participants who were not confident of their HFpEF diagnosis or were not diagnosed with it at all. This is consistent with other findings showing that heart failure still lacks adequate self-awareness in older adults, with only 26% of sufferers self-identifying their diagnosis, even after objective affirmation [33]. Participants who were uncertain or unaware of their HFpEF diagnosis showed worse functional status than those who were aware, which may be attributed to a lack of self-management and underdiagnosis. Previous studies support this, showing that low diagnostic awareness of heart failure is associated with lower clinical status and higher functional impairment, likely due to delayed intervention and poorer engagement in managing their health [34]. Patients who are unaware of their diagnosis may not have had the benefit of early treatment or advice on managing symptoms effectively, leading to more pronounced functional limitations and poorer symptom burden. Nevertheless, one of the interesting findings of our studies was that participants with knowledge of their HFpEF diagnosis reported a poorer quality of life compared to those who were uninformed or unsure of their diagnosis. This paradoxical outcome can be justified in several ways. The diagnosis awareness may result in heightened illness and symptom awareness, commonly accompanied by a greater sense of severity and mental load. This is consistent with the research indicating that quality of life tends to deteriorate following a heart diagnosis, as a result of the emotional and psychological toll of coping with a chronic condition, as well as the stress of treatment [35]. The possibility exists that a diagnosis may lead to a more accurate recognition of functional decline and symptoms, resulting in a fairer evaluation of quality of life. Conversely, more unaware people may be less conscious of their disease, and thus, claim a better life quality, potentially because they underrate symptoms or fail to realize the severity of their health problems. This demonstrates a paradox of diagnostics, where higher levels of awareness may correlate with a lowered self-reported quality of life, not due to the condition itself but rather the psychosocial effects and fear of disease progression that often accompany an established diagnosis.

In our research, impaired functional status and diminished quality of life were significant predictors of suspected cardiac amyloidosis compared with normal HFpEF. These results are aligned with the past data on patients of AL amyloidosis, where functional status and quality of life were associated with disease severity and adverse outcomes [36].

Older people in our study had a much higher prevalence of taking medication to treat heart disease, including beta-blockers and diuretics. Nonetheless, previous studies have demonstrated that, despite a higher disease burden, non-hospitalized adults aged over 75 have lower rates of cardiac medication use, supported by findings and evidence, raising the prospect of underutilization compared to the recommended clinical guidance [37]. This discrepancy could be due to factors such as underdiagnosis, medication hesitancy, or barriers to healthcare access, which might contribute to a lack of timely intervention and poorer outcomes for older individuals. In our study, older participants were more likely to report being unsure about the family history of heart disease, whereas middle-aged participants showed greater awareness. This finding is consistent with previous studies, which illustrate how older individuals tend to provide family health history on a selective basis, based on perceived importance and emotional attachment, thereby creating a knowledge gap later in life [38]. The lack of awareness among older individuals could delay intervention or lead to missed opportunities for early detection of heart disease risk, which might otherwise be managed with targeted treatments. These findings underscore the importance of improving healthcare interventions in older adults. While older individuals may use more heart-related medications, the potential underutilization in the oldest group emphasizes the need for targeted education and adherence support. Furthermore, increasing awareness of family health history among older adults could significantly enhance early detection of heart disease and its related risks. Ensuring comprehensive family history intake and improving medication adherence could ultimately lead to better health outcomes for this population.

Limitations

This study has a few limitations. To begin with, its cross-sectional design constrains its capacity to make causal inferences regarding the connection between red flag symptoms and functional or quality of life outcomes. Second, the research relied solely on self-reporting and observation, with no confirmatory tests used to validate the data, such as cardiac MRI, bone scintigraphy, or endomyocardial biopsy, making it prone to misclassification. Although it is valuable to use the red flag checklist as a screening mechanism, it has not been psychometrically validated. Third, the use of convenience sampling from cardiology departments in Lahore weakens the generalizability of results to unrestricted areas or medical facilities. While this method facilitated participant recruitment, it may not fully represent the wider population of heart failure patients. Efforts were made to recruit from multiple healthcare sites in Lahore, but future studies using random sampling methods could improve generalizability. There was also a possibility of recall bias regarding the items concerning the symptoms and medical history. Finally, the study did not look deeper into the possible impact of medication adherence, socioeconomic factors, and other comorbidities, which may additionally influence the identified relationships.

Future directions

Future studies should focus on confirming the red flag checklist utilized in the present study, either via longitudinal or diagnostic accuracy studies, preferably against verified cases of cardiac amyloidosis. The addition of biomarkers, echocardiography, and advanced imaging techniques may also enhance the precision of screening and expand the diagnostic route, providing a deeper understanding of early cardiac involvement. Echocardiographic vigilance, in particular, is critical in detecting subtle myocardial changes that may not be readily apparent through other diagnostic measures, making it an invaluable tool for early identification of amyloid cardiomyopathy. Multicenter studies in different areas of Pakistan or South Asia, such as large-scale studies, would contribute not only to the enhancement of the external validity of the research but also allow for comparing the results in various clinical settings. In addition, qualitative studies that would enable the determination of patients’ level of awareness, health-seeking behaviour, and issues that prevent early diagnosis in cardiac amyloidosis would be valuable in augmenting quantitative data. Lastly, future intervention research may be used to determine whether symptom-based screening as the first line of referral leads to better outcomes by initiating the proper course of treatment earlier.

## Conclusions

This cross-sectional study has demonstrated a significant relationship between an increased burden of red flag symptoms suggestive of cardiac amyloidosis and worsened functional status and quality of life in patients with symptoms of heart failure. Nevertheless, it is worth noting that no diagnostic tests were involved, and the results can be viewed as correlational rather than causal. These findings indicate that a symptom-based screening tool might serve as a potentially effective first step to targeting patients with characteristics related to cardiac amyloidosis (i.e., in the general population of HFpEF patients). Nonetheless, this screening tool is not diagnostic, as it still requires future prospective validation and confirmatory diagnostic tests to determine its clinical usefulness. As the study is based on an observational design, it is essential to note that the identified relationships are merely suggestive and cannot be considered definitive until future studies replicate the results and assess their clinical implications.

## Appendices

**Table 10:** Kansas City Cardiomyopathy Questionnaire (KCCQ-12).

Items	Responses
Over the past 2 weeks, how much were you limited by heart failure (shortness of breath or fatigue) in your ability to shower or bathe?	-
Over the past 2 weeks, how much were you limited by heart failure in your ability to walk 1 block on level ground?	-
Over the past 2 weeks, how much were you limited by heart failure in your ability to hurry or jog (as if to catch a bus)?	-
Over the past 2 weeks, how many times did you have swelling in your feet, ankles, or legs when you woke up in the morning?	-
Over the past 2 weeks, on average, how many times has fatigue limited your ability to do what you wanted?	-
Over the past 2 weeks, on average, how many times has shortness of breath limited your ability to do what you wanted?	-
Over the past 2 weeks, how often did you sleep sitting up in a chair or with at least 3 pillows to prop you up due to shortness of breath?	-
Over the past 2 weeks, how much has your heart failure limited your enjoyment of life?	-
If you had to spend the rest of your life with your heart failure as it is right now, how would you feel about this?	-
Over the past 2 weeks, how much has your heart failure limited your participation in hobbies or recreational activities?	-
Over the past 2 weeks, how much has your heart failure limited your ability to work or do household chores?	-
Over the past 2 weeks, how much has your heart failure limited your ability to visit family or friends outside your home?	-
